# Emerging Metal Additive Manufacturing for Individualized Dental Therapies: A Narrative Review

**DOI:** 10.3390/dj13090424

**Published:** 2025-09-15

**Authors:** Peng Chen, Taishi Yokoi, Ying-Sui Sun, Huiyong Yang, Hiroyasu Kanetaka

**Affiliations:** 1 Liaison Center for Innovative Dentistry, Graduate School of Dentistry, Tohoku University, 4-1 Seiryo-machi, Aoba-ku, Sendai 980-8575, Japan; 2 Laboratory for Biomaterials and Bioengineering, Institute of Integrated Research, Institute of Science Tokyo, 2-3-10 Kanda-Surugadai, Chiyoda-ku, Tokyo 101-0062, Japan; yokoi.taishi.bcr@tmd.ac.jp; 3 School of Dental Technology, College of Oral Medicine, Taipei Medical University, Taipei 111031, Taiwan; yingsuisun@tmu.edu.tw; 4 School of Medicine, Huaqiao University, 269 Chenghua North Road, Quanzhou 362021, China; baiqingyang_78@163.com; 5 Division of Orthodontics and Dentofacial Orthopedics, Graduate School of Dentistry, Tohoku University, 4-1 Seiryo-machi, Aoba-ku, Sendai 980-8575, Japan; 6 Division of Advanced Dental Science and Technology, Graduate School of Biomedical Engineering, Tohoku University, 6-6-12, Aramaki-aza-Aoba, Aoba-ku, Sendai 980-8579, Japan

**Keywords:** additive manufacturing, personalized dental treatments, laser powder bed fusion, titanium alloy, digital dentistry, custom dental prosthetics

## Abstract

Metal additive manufacturing (AM) techniques, particularly laser powder bed fusion, are being increasingly recognized not as brand-new technologies, but as emerging technologies with their recent advancements—such as the development of optimized alloys, seamless digital workflow integration, and applications in patient-specific prostheses. With the rise in patient-specific approaches in dentistry, clinicians are seeking customized devices that precisely match individual anatomical and functional needs. AM offers various advantages, such as the fabrication of complex geometries directly from digital designs, enhanced clinical precision, reduced material waste, and simplified manufacturing workflow, and hence can uniquely address these demands. Recent advancements in AM techniques have led to the development of titanium and cobalt–chromium alloys with improved mechanical properties, corrosion resistance, and biological compatibility. These alloys show great potential for clinical applications. Additionally, AM enables precise control over the microstructures and surface topographies of these alloys during fabrication, facilitating their optimized integration with biological tissues. This mini review summarizes recent advancements in metal AM technologies relevant to personalized dentistry, highlights key material developments, discusses current clinical applications, and identifies key challenges such as high cost, materials limitations, and regulatory hurdles, and highlights future opportunities including multi-materials AM, smart implants, and AI-driven optimization for fully integrated, digitally driven personalized dental care.

## 1. Introduction

Driven by the growing demand for patient-specific solutions tailored to individual anatomical and functional requirements, personalized dental treatments have emerged as a transformative paradigm in modern dentistry. Unlike traditional methods, which primarily rely on standardized components and techniques, personalized dentistry emphasizes precision, adaptability, and biological integration tailored to the requirements of each patient [1,2,3]. Such approaches are particularly relevant in complex clinical cases involving congenital anomalies, extensive edentulism, or post-traumatic deformities, in which off-the-shelf components often fail to provide satisfactory esthetic and functional outcomes. Personalized treatments offer a way to overcome these limitations by delivering devices that closely align with patient-specific conditions, both morphologically and biomechanically. This approach necessitates dental components that not only conform to the anatomical and functional requirements of each patient but also exhibit predictable mechanical properties, biological compatibility, and efficient integration with digital workflows [4,5]. Moreover, the demand for such customization is accelerating along with the growth of digital dentistry, which has revolutionized diagnostic imaging, design planning, and data-driven manufacturing. In this context, metal additive manufacturing (AM) is rapidly becoming a key enabler of personalized treatment strategies by offering seamless integration into these digital systems. Although polymers and ceramics are increasingly used in dentistry, metallic materials remain indispensable in clinical applications requiring superior mechanical strength, durability, and long-span stability—such as implant frameworks and large prostheses—which cannot be fully replaced by alternative materials.

Owing to their ability to fabricate complex geometries directly from digital models, metal AM techniques, particularly laser powder bed fusion (L-PBF) technologies, such as selective laser melting (SLM) and direct metal laser sintering (DMLS), have emerged as key enablers of personalized dentistry [6,7]. The inherent flexibility of metal AM enables the efficient fabrication of patient-specific dental devices, ranging from customized implant frameworks and orthodontic appliances to surgical guides, offering improved accuracy, reduced production time, and minimal material waste [3,4,6,8]. Unlike conventional subtractive methods that require extensive manual input and intermediate tooling, AM enables clinicians and dental technicians to directly translate virtual treatment plans into functional restorations with high-dimensional fidelity. This capability significantly reduces the back-and-forth iterations that often occur in traditional workflows, thereby enhancing efficiency and reproducibility.

Recent advancements in AM-compatible metal alloys, particularly titanium and cobalt–chromium alloys (Co–Cr alloys) systems [9], have significantly enhanced the clinical feasibility of AM in dentistry [2,10,11]. These alloys provide not only robust mechanical properties and excellent corrosion resistance but also favorable biological responses and hence are suitable for safe and effective long-term integration with oral tissues [12,13,14]. Although several prior reviews, such as those by Revilla-León et al. [6], have broadly summarized metal AM technologies in implant dentistry, the present review focuses specifically on the integration of AM into personalized treatment workflows. In contrast to general overviews, this work emphasizes patient-specific customization, material-process optimization, and synergy between AM techniques and digital design tools, highlighting their role in achieving clinically relevant, individualized outcomes. Additionally, such materials can be processed under carefully controlled AM parameters to achieve tailored surface morphologies and porosities, which are known to influence early-stage tissue responses, such as fibroblast adhesion, osseointegration, and soft tissue sealing. These effects are particularly critical in the peri-implant region, where microleakage and bacterial colonization can lead to long-term complications. Moreover, AM processes enable precise control over the microstructural characteristics and surface topographies of these alloys, enabling clinicians to optimize the interface between the dental components and the surrounding biological environment [15,16,17]. This level of control opens new possibilities for the fabrication of hybrid dental devices that combine rigid internal structures with biofunctionalized outer layers, thereby maximizing the mechanical reliability while supporting favorable cellular interactions.

According to recent market research [18], the global dental three-dimensional (3D) printing market was valued at approximately USD 3.1 billion in 2023 and is projected to grow at a compound annual growth rate (CAGR) of 26.4% from 2024 to 2030, reaching nearly USD 15.9 billion by the end of the decade. This rapid growth highlights the significant commercial potential and accelerating clinical adoption of metal AM in personalized dentistry, underscoring the importance of staying abreast of technological developments and their clinical implications. It also reflects a broader shift in the dental industry toward value-based, patient-centered care models that prioritize long-term outcomes, efficiency, and personalization over volume-based service delivery.

This mini review summarizes the recent progress in metal AM technologies relevant to personalized dental treatments. It highlights key advances in material development for dental treatments, discusses current clinical applications and outcomes, and identifies ongoing challenges and future opportunities for fully integrated, digitally driven personalized dental care.

## 2. Recent Advances in Metal AM for Dental Applications

Over the past decade, metal AM has experienced rapid advancements [19] in processing technologies, powder materials, and digital integration, with several key developments reported in recent years (2022–2025) also included in this review. Compared with conventional casting and milling, AM-fabricated prostheses generally achieve higher dimensional fidelity, with deviations typically within tens of microns, which is clinically acceptable for long-term function. SLM and DMLS, which enable the layer-by-layer fabrication of complex geometries with high precision (Table 1), are the most widely used techniques in dentistry. These technologies allow for the direct conversion of three-dimensional CAD data into physical structures with a micrometer-level resolution, which is particularly valuable for fabricating anatomically accurate dental components. The absence of intermediate molds or subtractive steps further shortens the production time and reduces contamination risk.

**Figure 1:**
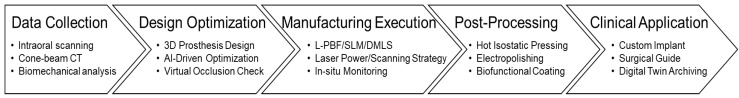
Digital workflow of metal additive manufacturing in personalized dentistry.

A schematic overview of the digital workflow of metal AM in dentistry is provided in Figure 1, illustrating the process from intraoral scanning, CAD design, and AM fabrication to clinical application.

A critical advancement that has led to the widespread adoption of AM in dentistry is the development of AM-optimized alloys. Titanium alloys, notably Ti-6Al-4V and emerging β-type titanium alloys, have gained significant attention for dental applications owing to their high strength-to-weight ratio, excellent corrosion resistance, and favorable biocompatibility [1,20,21,22]. Recent studies on Ti alloys prepared using graphene oxide (GO)-modified powders have demonstrated that carbon-supersaturated β-Ti alloys fabricated by L-PBF exhibit ultrafine grain structures and achieving tensile strength exceeding 1100 MPa while retaining excellent cytocompatibility [23]. This grain refinement effect can be attributed to the rapid solidification conditions intrinsic to L-PBF processes, which suppress grain growth and favor heterogeneous nucleation. Additionally, the incorporation of GO during powder preparation may contribute to carbon solute strengthening and thermal stability, providing a dual benefit in terms of mechanical enhancement and microstructure control. These results highlight the potential of using nanoscale additives (during powder preparation) as a promising strategy for the development of high-performance AM materials. Furthermore, titanium-based materials exhibit favorable elastic moduli that can be tuned via design parameters or internal structure modifications, allowing for better mechanical compatibility with the alveolar bone. This tunability is especially advantageous in reducing stress shielding and promoting peri-implant bone remodeling. Similarly, Co–Cr alloys fabricated via AM remain vital in dental AM applications, particularly for prosthetic frameworks, owing to their robust mechanical properties and corrosion resistance. Advancements in alloy refinement processes, such as reducing impurities and optimizing powder characteristics, have significantly improved the quality and reliability of AM-fabricated Co–Cr dental components. Compared to titanium, Co-Cr alloys offer higher stiffness and wear resistance, making them suitable for large-span prostheses or removable frameworks. Moreover, modern AM systems enable better control over porosity and microsegregation during the solidification of Co-Cr alloys, reducing the risk of internal defects and improving overall durability.

Corrosion resistance, a crucial factor in the long-term success of dental restorations, has also benefited from recent advances in AM. A recent study revealed that L-PBF-fabricated 316 L stainless steel exhibits significantly improved pitting and crevice corrosion resistance compared to its conventional counterparts [24]. This improvement is independent of the crystallographic texture and grain boundary density of the alloy and can be attributed to its rapid solidification and refined microstructures and the suppression of inclusion formation during the AM process. Such enhanced corrosion behavior plays a pivotal role in minimizing metal ion release in the oral environment, thereby reducing the risk of local inflammation, allergic reactions, and long-term systemic effects. As a result, AM-fabricated components provide a more stable and biocompatible solution for intraoral applications, particularly in complex or long-term restorations. These findings highlight the intrinsic advantages of metal AM not only for shaping complex geometries but also for ensuring enhanced chemical stability and biological safety in clinical applications.

Another notable advancement in AM is the precise control of the surface and internal microstructures of the resulting alloys, which is achieved by optimizing the AM process parameters [25,26]. Process parameters, such as the laser power, scanning strategies, hatch spacing, and layer thickness, are adjusted to achieve customized material properties, including porosity and elastic modulus, to better match the mechanical behavior of natural dental tissues. This high degree of control allows for the fabrication of gradient structures within a single build, where internal regions can be made denser for strength, and external layers more porous to promote tissue integration. Additionally, micro- and nanoscale surface roughness achieved by tuning the scanning strategies can positively influence early cell adhesion and osseointegration.

Tailored surface textures of AM-fabricated alloys at the micro- and nanoscale levels offer enhanced biological integration, reduced healing times, and improved clinical outcomes in implant dentistry.

Furthermore, post-processing strategies [27,28,29,30,31], such as hot isostatic pressing (HIP) [32], heat treatments, and advanced surface finishing techniques (e.g., electropolishing and chemical etching), have undergone significant advancements. These techniques improve the microstructural characteristics, fatigue resistance, and surface biocompatibility of AM-produced metal components, thus addressing their traditional limitations and facilitating their clinical adoption. Electropolishing, for instance, not only reduces surface roughness but also helps remove process-induced contaminants, thereby enhancing cleanliness and minimizing bacterial adhesion. Similarly, HIP can eliminate internal voids and residual stress, further increasing mechanical stability under cyclic loading.

Metal AM is uniquely suited for seamless integration into the digital dentistry workflow. Digital intraoral scanning, combined with sophisticated CAD/CAM software, facilitates rapid and accurate transfer of patient-specific data directly to AM production units. Integration of artificial intelligence (AI) in design optimization is driving new advancements in automating and refining patient-specific solutions. For example, AI algorithms can suggest optimal implant positioning based on anatomical landmarks, automatically generate support structures, or propose build orientations that minimize distortions. These functions not only accelerate the design process, but also enhance precision and repeatability. This approach streamlines treatment planning, enhances clinical accuracy, and reduces the diagnosis-to-delivery time, significantly enhancing patient-centered care.

Collectively, these advancements in materials, processing strategies, and digital integration underscore the transformative potential of metal AM in personalized dentistry. However, continued research and development are essential to overcome current limitations and drive future innovations.

## 3. Clinical Applications of Metal AM in Personalized Dental Treatments

The increasing need for patient-specific solutions across therapeutic areas, such as implantology, prosthodontics, orthodontics, and maxillofacial surgery, has led to a rapid growth in the clinical adoption of metal AM in personalized dentistry. Metal AM technologies, particularly L-PBF, facilitate unprecedented customization and precision, significantly enhancing patient outcomes and clinical workflows [3]. This level of customization enables practitioners to tailor treatment plans according to individual anatomical constraints, occlusal relationships, and aesthetic considerations. As a result, AM is helping shift dentistry from a technician-driven process to a data-driven, clinician-directed discipline.

A wide range of clinical applications has benefited from the unique capabilities of metal AM (Table 2). In dental implantology, for instance, AM enables the direct fabrication of customized implant-supported frameworks and patient-specific abutments. These customized abutments can be tailored to match patient-specific gingival contours and emergence profiles, helping to reduce peri-implant complications and promote soft tissue health [33]. Compared to prefabricated abutments, custom AM designs can better distribute stress around the implant site and reduce microgaps at the implant–abutment interface, which are critical for maintaining long-term peri-implant tissue stability. Similarly, AM-fabricated implant frameworks can precisely accommodate individual anatomical and occlusal requirements, minimizing chairside adjustments and enhancing long-term prosthetic stability. This is particularly advantageous in full-arch restorations or complex edentulous cases where a precise passive fit is essential to avoid biomechanical complications. In practice, AM-fabricated implant frameworks typically demonstrate dimensional deviations within tens of microns, which is considered clinically acceptable, and recent retrospective studies report survival rates of AM implants exceeding 95% at one year, supporting their feasibility in routine dentistry.

Metal AM is also transforming prosthodontics by enabling the direct digital fabrication of complex prosthetic structures, including crowns, bridges, partial dentures, and full-arch frameworks [34,35,36,37,38]. By using intraoral scans and CAD designs, AM reduces the manufacturing time, minimizes manual fabrication errors, and ensures high-dimensional accuracy and consistency [39]. Furthermore, this digital fabrication process enhances reproducibility, allowing dental laboratories to replicate identical frameworks in future revisions or replacements with minimal effort. Moreover, the flexibility of metal AM enables the functional grading of the properties of prosthesis materials, thereby optimizing their mechanical performance and patient-specific biological responses. This approach is especially beneficial when adapting the stiffness or porosity across different regions of a prosthesis to match the functional demands of the underlying tissues.

In orthodontics, metal AM has facilitated the production of customized appliances [40,41], such as patient-specific brackets, arch wires, and skeletal anchorage devices. These components are precisely tailored to patient anatomy and treatment objectives, enabling accurate replication of digitally planned tooth movements. Traditional orthodontic appliances often require manual adaptation and adjustment, whereas AM-fabricated devices provide a more precise starting point and can significantly reduce chair time. These advantages of metal AM render orthodontic treatments more predictable, efficient, and comfortable than conventional treatments [42].

Maxillofacial surgery is another clinical area that significantly benefits from metal AM [43,44]. Customized surgical guides produced via AM enhance surgical planning and precision while reducing operative complexity and procedure duration. They offer intraoperative accuracy by defining cutting planes or implant placement trajectories directly from the preoperative imaging data. Additionally, AM-fabricated titanium fixation plates and patient-specific reconstructive scaffolds offer enhanced anatomical fit, mechanical stability, and biological integration, contributing to faster healing and better postoperative outcomes [45]. In cases of mandibular or maxillary reconstruction, the use of patient-specific titanium plates helps to restore facial symmetry and occlusal function while minimizing the need for intraoperative bending or plate modification.

Moreover, the integration of AM with digital tools, including intraoral scanning, cone-beam computed tomography, CAD/CAM software, and AI-driven optimization, has significantly streamlined clinical workflows. This digital integration allows efficient collaboration among clinicians, laboratories, and patients, facilitating rapid prototyping, real-time design adjustments, and the delivery of highly personalized care [46,47]. For example, interdisciplinary teams can remotely review and revise implant frameworks or surgical plans using cloud-based platforms, reducing turnaround time and improving communication. Furthermore, AM enables traceability and documentation of each prosthesis or device, which enhances quality control and supports regulatory documentation.

Despite these advances, the adoption of metal AM in personalized dentistry still faces several challenges, such as regulatory compliance, the lack of standardized clinical protocols, and the need for long-term outcome validation. To fully realize the transformative potential of metal AM in personalized dentistry, continued research, rigorous clinical evaluation, and establishment of comprehensive regulatory frameworks are essential. Outcome-based metrics and multicenter studies are necessary to establish evidence-based guidelines and build clinical confidence in AM-based devices.

Collectively, these diverse clinical applications highlight the significant potential of metal AM technologies in delivering precise and individualized dental care. Ongoing innovations and research are expected to further integrate AM into routine clinical practice, establishing it as a cornerstone of future personalized dentistry. In addition to specific clinical examples, the global adoption of AM in dentistry has been steadily increasing. Dental laboratories and clinics are progressively incorporating AM technologies, particularly for implant frameworks and removable prostheses, reflecting a growing demand for efficiency, precision, and patient-specific customization that conventional methods often cannot achieve.

To aid readers in navigating the relevant literature discussed in this review, Table 3 provides a summary of representative studies on metal AM technologies and their clinical- or material-specific contributions in dentistry.

As summarized in Table 3, representative studies highlight both material-level innovations (e.g., β-Ti alloy with nanoscale additives, improved corrosion resistance in stainless steel) and clinical applications (e.g., customized abutments, reconstructive plates). Together, these examples demonstrate the dual progress in fundamental materials development and practical clinical integration.

## 4. Challenges and Future Perspectives

Although metal AM has significantly advanced personalized dental treatments, several key challenges, such as ensuring consistency in material performance, establishing regulatory standards, improving cost efficiency, and validating long-term clinical outcomes, must still be addressed to fully realize its clinical potential [48,49,50]. Addressing these issues is essential to realize the widespread clinical adoption of AM and to ensure predictable and reliable outcomes in clinical practice. The interdisciplinary nature of AM-based dentistry—spanning materials science, clinical dentistry, and digital engineering—also necessitates a more integrated approach to problem-solving and implementation.

One of the primary challenges associated with AM-fabricated dental devices is ensuring consistent material quality and mechanical performance. Variations in the processing parameters, such as the laser energy, scanning strategy, and powder characteristics, can significantly affect the microstructure and mechanical properties of AM-fabricated components [51]. These inconsistencies may lead to local defects, such as porosity, incomplete fusion, or residual stress, which can compromise fatigue resistance or cause long-term deformation under masticatory loads. Thus, future research should prioritize the development of standardized processing guidelines and robust quality assurance protocols to minimize variability, enhance reproducibility, and ensure reliable clinical performance. Machine learning-based monitoring systems, for instance, could be employed to identify suboptimal build conditions in real time, enabling defect mitigation before final production.

Inadequate regulatory standardization remains a significant barrier to the clinical adoption of AM-fabricated devices. Owing to the complexity and personalized nature of these devices, traditional regulatory frameworks may fall short in addressing the unique patient-specific design and fabrication aspects of these devices. Current regulations are largely adapted from conventional manufacturing paradigms and may not account for the variability and customization inherent in AM workflows. Thus, establishing comprehensive and standardized regulatory guidelines tailored specifically for AM is essential to promote broader clinical adoption and streamline approval processes [52,53,54,55,56]. The international harmonization of these standards will also be crucial for global market acceptance, particularly given the cross-border nature of digital design and fabrication services.

Cost-effectiveness is also a significant challenge in the widespread implementation of metal AM in dentistry. The high initial investment required for equipment, specialized powders, and post-processing tools may limit the accessibility of metal AM, especially for smaller clinics or laboratories [57,58]. In addition, the labor cost and time associated with training personnel in AM-specific design and software tools add to the operational burden. To address this, efforts should focus on developing cost-effective AM systems, improving powder reuse strategies, optimizing resource utilization, and minimizing post-processing costs while maintaining product quality and clinical efficacy [59,60]. Collaborative AM networks or centralized fabrication hubs may help distribute costs while maintaining high-quality production standards. For example, AM workflows can reduce material waste compared with subtractive milling (often >50% waste in conventional processes), but the initial equipment cost and need for specialized training remain significant barriers.

Moreover, long-term clinical validation of AM-produced dental devices remains relatively limited. While short- to medium-term clinical outcomes are promising, extensive longitudinal studies are needed to assess durability, biological integration, and sustained clinical performance [61]. Factors such as micromotion at the implant–abutment interface, corrosion in complex oral environments, and biofilm formation over time must be systematically studied to confirm the long-term viability of AM-fabricated components. Comprehensive clinical trials and systematic follow-up studies are essential for establishing the long-term safety, effectiveness, and reliability of the metal AM.

Several promising opportunities exist to further enhance the role of metal AM in personalized dentistry. One particularly promising area is the integration of AI and machine learning into digital design workflows. AI-driven systems can analyze large clinical databases to optimize patient-specific device geometries and predict the biological responses of AM-fabricated devices while improving their mechanical properties. Such intelligent systems could also assist in presurgical simulations, failure risk predictions, and automated generation of treatment alternatives, all contributing to greater clinical precision. This integration can significantly enhance the accuracy, efficiency, and predictability of personalized dental treatments.

Another promising direction is the development of next-generation alloy systems specifically engineered for AM. These advanced alloys offer improved biological compatibility, enhanced mechanical properties, and tailored degradation behaviors, thus demonstrating their huge potential for clinical applications. In addition to binary and ternary titanium systems, high-entropy alloys and bioresorbable metallic systems are gaining interest as future candidates for functionally adaptive dental components. Incorporating functional gradients (such as variations in material composition, porosity, or mechanical properties) within a single AM-fabricated component could enable unprecedented levels of customization and performance optimization. Such gradients could allow a prosthesis to be simultaneously rigid in load-bearing regions and porous in soft-tissue contact areas, achieving both strength and bioactivity within one design.

Additionally, the concept of digital twins, which involves virtual replicas of patient-specific dental treatments stored and continuously updated in digital archives, holds transformative potential for clinical practice. These digital archives enable precise long-term monitoring, facilitate efficient repair or replacement of devices, and generate valuable longitudinal data for research and continuous treatment optimization. With increasing access to cloud-based patient records and wearable sensors, digital twins may evolve into real-time diagnostic platforms that continuously inform personalized dental care.

Another important limitation lies in the training and digital literacy of dental professionals. Effective use of AM-based workflows requires clinicians and technicians to be proficient in digital scanning, CAD modeling, and AM-specific design principles. Without adequate training, the potential benefits of AM may not be fully realized in clinical practice.

In conclusion, while significant challenges persist, ongoing research and technological advancements have positioned metal AM as a foundational pillar in the future of personalized dentistry. Bridging the gap between material innovation, clinical validation, and economic scalability is the key to unlocking its full potential. By addressing current limitations and leveraging the emerging opportunities in digital integration, materials science, and intelligent design, metal AM is poised to continue evolving, offering improved clinical outcomes and patient care in personalized dental treatments.

## 5. Conclusions

Metal AM is redefining personalized dental treatments by enabling the direct fabrication of customized, anatomically precise, and functionally optimized devices. Its seamless integration with digital workflows, ability to produce complex geometries, and adaptability to patient-specific requirements have demonstrated significant advantages across diverse range of clinical applications, including implantology, prosthodontics, orthodontics, and maxillofacial surgery. Compared with conventional casting or milling, AM-fabricated devices generally require fewer manual adjustments, reduce overall treatment time, and provide improved anatomical accuracy, thereby enhancing clinical efficiency.

Recent advancements in AM-compatible alloys, microstructural control, and post-processing techniques have significantly improved the mechanical and biological performance of metal AM components. Moreover, the integration of artificial intelligence and digital design tools is accelerating the transition toward fully individualized, data-driven dental care.

Despite these promising developments, several challenges—including high equipment cost, lack of standardized protocols, and limited long-term clinical validation—must be addressed. At the same time, emerging opportunities such as multi-material AM, functionally graded implants, digital twins, and AI-driven optimization highlight the transformative potential of metal AM in dentistry. Ultimately, metal AM is poised to become a cornerstone technology in precision dental care, enabling highly customized and clinically effective treatment solutions.

Ultimately, metal AM is poised to become a cornerstone technology in the future of precision dental care, enabling a new era of patient-centered, efficient, and highly customized treatment solutions.

## Figures and Tables

**Table 1:** Comparison of Conventional Methods and AM for Fabricating Metal Dental Prostheses.

Aspect	Conventional Methods	AM
Manufacturing method	Casting/Milling	SLM/DMLS/EBM
Material waste	High (due to subtractive process)	Low (layer-by-layer building)
Design complexity	Limited by tooling and molds	High (lattice, graded porosity, etc.)
Customization level	Stock-based	Fully patient-specific from digital data
Production time	Long (multi-step workflows)	Short (digital-to-print in one flow)
Mechanical properties	Reliable but limited shape control	Tailored via printing parameters
Biocompatibility enhancement	Relies on coating or polishing	Post-processable with advanced surfaces
Common materials	Titanium alloys, Co-Cr alloys	Titanium alloys, Co-Cr, new alloys

Note: The comparison emphasizes conventional workflows that are more manually intensive; modern precision casting and milling techniques may achieve higher efficiency in some contexts, but AM uniquely offers seamless digital integration and full patient-specific customization.

**Table 2:** Clinical Applications of AM for Personalized Dentistry.

Application	Clinical Benefit	AM-Specific Feature
Custom implant abutments	Improved emergence profile and fit	Digital workflow, high-resolution fabrication
Full-arch bars/ implant frameworks	Adaptation to complex edentulous cases	Single-piece fabrication with anatomical precision
Anatomically accurate crowns and bridges	Enhanced esthetics and occlusal harmony	Data-driven design with occlusion matching
Mandibular/maxillary reconstruction plates	Precise anatomical conformity post-trauma	3D anatomical scanning + direct metal printing
Functionally graded prostheses	Tailored stiffness/load distribution	Variable porosity and geometry in one build
AI-assisted prosthesis design	Predictive design optimization	Integration of clinical datasets and AI models
Digital twin archiving	Quick remakes, consistent patient data use	Reusable CAD models for future use

**Table 3:** Summary of Representative Studies on Metal Additive Manufacturing in Dentistry.

Material	AM Method	Application	Key Findings	First Author (Year)	Reference Number
*β*-Ti alloy (GO-modified)	L-PBF	Implantology	Ultrafine grains, >1100 MPa strength, cytocompatibility	Dong (2024)	[23]
316 L Stainless Steel	L-PBF	Corrosion analysis	Enhanced pitting and crevice corrosion resistance	Tsutsumi (2021)	[24]
Titanium	SLM	Custom implants	Precise fit to gingival contour and emergence profile	Chen (2014)	[33]
Co-Cr-Mo alloy	AM	Prosthodontics	Stable ion release, physiological corrosion behavior	Vasylyev (2025)	[34]
Stainless Steel	3D Printing	Orthodontics	Mechanical evaluation of printed appliances	Dragos (2024)	[40]
Titanium	AM	Maxillofacial surgery	Subperiosteal implant for maxillary reconstruction	De Riu (2025)	[43]
Titanium (AM implants)	AM	Implantology	1-year retrospective clinical evaluation	Mafra (2024)	[11]
Titanium	AM	Mandibular support	Custom fixation plates reduce fracture risk	Onică (2025)	[45]
Various	Digital Integration	Virtual articulators	Improved digital precision and design workflow	Lobo (2025)	[46]
Metal (General)	AM	Implant dentistry	Review of AM technologies and clinical status	Revilla-León (2020)	[6]

## Data Availability

Not applicable.
